# Modulation of benzofuran structure as a fluorescent probe to optimize linear and nonlinear optical properties and biological activities

**DOI:** 10.1007/s00894-020-04539-6

**Published:** 2020-09-19

**Authors:** Przemysław Krawczyk

**Affiliations:** Collegium Medicum, Faculty of Pharmacy, Department of Physical Chemistry, Nicolaus Copernicus University, Kurpińskiego 5, 85-950 Bydgoszcz, Poland

**Keywords:** Linear and nonlinear optical properties, Bioimaging, Conjugation with proteins, Biological activities, Toxicology, Solvatochromism, Benzofuran

## Abstract

The study presents the influence of structure modulation by introducing selected donor and acceptor substituents on optical properties of benzofuran used in biological imaging. As the starting form, 2-(5-formylbenzofuran-2-yl)acetamide described experimentally was used. This molecule contains an aldehyde group as reactive site, through which conjugation with protein occurs. Structure modulation was carried out by attaching additional electron-donating and electron-withdrawing substituents to the amino group, namely -NH_2_, -NHCH_3_, -NO_2_, -OH, and -OCH_3_. Studies have shown that the -NH_2_, -NHCH_3_, -OH, and -OCH_3_ substituents do not induce a significant change in the position of maximum absorption and fluorescence relative to each other. They also do not change the parameters describing the nonlinear response. Only the presence of the -NO_2_ substituent results in significant solvatochromic shifts. Changing substituents also does not significantly affect the LD_50_ value, and all tested fluorescent probes should not be considered toxic to humans. Modulation of the benzofuran derivative structure also does not change the active center in which the biocomplex with the protein is formed. In each case, the conjugation takes place via LYS114. In addition, the study was prompted to analyze the linear and nonlinear optical properties of conjugates formed after the reaction with Concanavalin A.

Graphical abstract

**Electronic supplementary material:**

The online version of this article (10.1007/s00894-020-04539-6) contains supplementary material, which is available to authorized users.

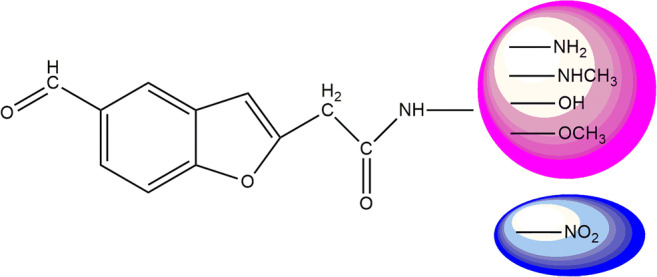

## Introduction

Benzofuran, because of its structure made of fused benzene and furan rings, can be classified as a heterocyclic organic compound. It is mainly utilized in the production of coumarone resins and it occurs naturally in the form of a colorless, oily liquid. Light oils obtained during coal tar distillation are the main source of this compound. There are several fields of application of benzofuran including organic synthesis, where it is used as a constituent for more complex systems, as well as material science. It is also widely found in many molecules that are biologically significant. Several different biological activities can be attributed to the derivatives of benzofuran. These include antiinflammatory, antioxidant, antitubercular, antiplasmodial, antitumor, antimicrobial, cytotoxic, enzyme inhibitory, HIV, and hepatitis C virus inhibitory activities [1–4]. The properties of benzofuran that are utilized in material science include on the other hand beneficial electrochemical behavior, thermal stability, high quantum yields, and blue-light emitting [5–7]. Also, their hole-transporting material properties [8, 9] make then useful for application as organic light-emitting diodes (OLED). Another interesting derivative is the benzofuran-naphthyridine, which can be characterized with high fluorescence and quantum yield with solvatochromic properties [10]. Fluorobenzofuran on the other hand is used as a high triplet energy host material in the design of green phosphorescent OLEDs [11]. Recently, there are ongoing efforts to utilize benzofuran hydrazones as potential scaffolds in the development of multifunctional drugs [12]. The use of molecular docking studies also suggested antimicrobial and antifungal activity of the probe molecules [13, 14]. Studies have also been carried out on the benzofuran derivative 2MBA in terms of its spectroscopic, quantum chemical, molecular docking, and drug likeness parameter properties. These studies suggested the pharmaceutical potential of the probe molecule [15, 16], while the molecular modeling studies on benzofuran derivatives indicate their potential as anticancer agents [17].

In the case of potential use of benzofuran derivatives as fluorescent probes, it is very important to study solvatochromic behavior. Such analysis should include an assessment of the polarity of the excited state, as they furnish information about the changes in electronic distribution and symmetry of the molecule in the excited state. The knowledge of the excited state dipole moment of the molecule would be helpful in explaining the nature of excited states, in describing the course of its photophysical/photochemical transformations, and in designing the nonlinear optical devices, and it allows one to judge the site of attack by electrophilic and nucleophilic reagents in photochemical reactions, etc. [18–28].

**Fig. 1:**
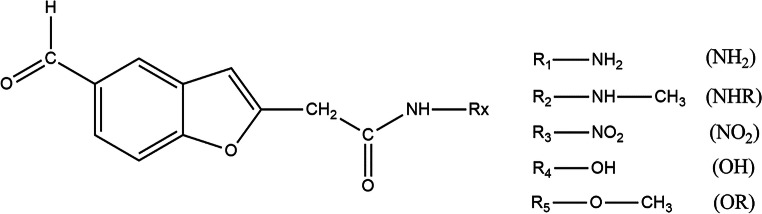
Structures of tested derivatives

Currently, there is very little information in the literature on the subject of benzofuran derivatives in the context of their linear and nonlinear (NLO) optical properties. Moreover, there is no data on conjugates of these dyes with proteins and the impact of structure modulation on spectral and NLO properties. Due to the frequent use of benzofuran derivatives in the pharmaceutical and medical industry, the paper presents a detailed analysis of the 2-(5-formylbenzofuran-2-yl)acetamide derivative. The analysis covers not only the molecule itself but also its derivatives resulting from the attachment of various electronodonor/acceptor substituents (Fig. 1). In addition, a thorough assessment of changes in the properties of the presented fluorescent probes after conjugation with the protein Concanavalin A (ConA) was performed. The choice of this derivative was not accidental. It is dictated by research carried out by Maridevarmath et al. [29], where a similar structure of 2-(5-methylbenzofuran-2-yl)acetohydrazide was analyzed. The differences consist in replacing the methyl group with an aldehyde group, through which the coupling with the protein occurs.

## Computational details

All geometrical parameters of investigated molecules in their ground (S_GS_) and excited states (S_CT_) were calculated using the density functional theory approach with the PBE0 functional implemented in the Gussian09 program package [30] with the TIGHT optimization threshold option and the 6-311++G(d,p) basis set. In order to verify that all the structures correspond to the minima on the potential energy surface, an analysis of Hessians was performed. The electronic properties were characterized by computations of the vertical absorption and emission spectra, which were obtained using the time-dependent density functional theory (TDDFT/PBE0) [31] and by including the state-specific (SS) corrected linear response (cLR) approach [32]. Due to the high compatibility of theoretical and experimental data [33–36], all spectroscopic calculations were performed using the PBE0 functional.

For the best consideration of the solvent impact on the fluorescence spectra, the ground state should be calculated with nonequilibrium solvation [37, 38]. This was taken into account by including the state-specific (SS) corrected linear response (cLR) approach [32] to the theoretical calculations. In the SS approach, the solvent dynamic polarizations are determined by the difference of the electron densities of the initial and final states [39–41].

The dipole moments and polarities of the charge-transfer state (S_CT_) were evaluated by numerical differentiation of the excitation energies (*E*) in the presence of an electric field *F* of 0.001 a.u. strength:

1 $$ \Delta  {\mu}_{g- CT}^i=\frac{E_{CT}\left(+{F}^i\right)-{E}_{CT}\left(-{F}^i\right)}{-2{F}^i}-\frac{E_g\left(+{F}^i\right)-{E}_g\left(-{F}^i\right)}{-2{F}^i} $$

where *i* stands for the Cartesian component of the dipole moment difference and *g* is the ground state (S_GS_). The isotropic average polarizability (〈*α*〉), polarizability anisotropy (Δ*α*), and first-order hyperpolarizability (*β*_vec_) were determined based on the Gaussian 09 program and defined as:

2 $$ \left\langle \alpha \right\rangle =\frac{\alpha_{xx}+{\alpha}_{yy}+{\alpha}_{zz}}{3} $$

3 $$ \Delta  \alpha =\sqrt{\frac{{\left({\alpha}_{xx}-{\alpha}_{yy}\right)}^2+{\left({\alpha}_{xx}-{\alpha}_{zz}\right)}^2+{\left({\alpha}_{yy}-{\alpha}_{zz}\right)}^2+6\left({\alpha}_{xy}^2+{\alpha}_{xz}^2+{\alpha}_{yz}^2\right)}{2}} $$

4 $$ {\beta}_{vec}={\sum}_{i=x,y,z}\frac{\mu_i{\beta}_i}{\left|\mu \right|} $$

where *β*_*i*_(*i* = *x*, *y*, *z*) is given by ${\beta}_i=\left(\frac{1}{3}\right){\sum}_{j=x,y,z}\left({\beta}_{ijj}+{\beta}_{jij}+{\beta}_{jji.}\right)$

The density differences were obtained at the PBE0/6-311++G(d,p) level and are represented with a contour threshold of 0.02 a.u. In these graphs, the blue (purple) zones indicate density decrease (increase) upon electronic transition. The charge-transfer parameters, namely the charge-transfer distance (*D*_CT_) and the amount of transferred charge (*q*_CT_), have been determined following a Le Bahers’ procedure [42]. The solvent effect on the linear and nonlinear optical properties has been taken into account using the Integral Equation Formalism for the Polarizable Continuum Model (IEF–PCM) [43, 44].

Experimentally, the two-photon absorption (TPA) can be obtained by the dissipation of the incident light, which for a single-beam 2PA experiment is twice the transition rate. In this case, the two-photon cross section of the degenerate process is written as [45–47]:

5 $$ {\upsigma}_{OF}^{(2)}=\frac{8{\pi}^3{\alpha}^2{\mathrm{\hslash}}^3}{e^4}\cdot \frac{\omega^2g\left(\omega \right)}{\Gamma_F/2}\left\langle {\delta}^{OF}\right\rangle $$

where *α* is a fine structure constant, *ω* is the frequency of absorbed photons (assuming one source of photons), *Γ*_F_ is the broadening of the final state (F) due to its finite lifetime, and g(*ω*) provides the spectral line profile, which often is assumed to be a *δ*-function and 〈*δ*^OF^〉 is the two-photon transition probability for the transition from the ground state to a final state.

In the case of a molecule absorbing two photons of the same energy in isotropic media, the degenerate 〈*δ*^OF^〉 in an isotropic medium using a linearly polarized laser beam given by [48]:

6 $$ \left\langle {\delta}^{OF}\right\rangle =\frac{1}{15}{\sum}_{ij}\left[{S}_{ii}^{OF}{\left({S}_{jj}^{OF}\right)}^{\ast }+2{S}_{ij}^{OF}{\left({S}_{ij}^{OF}\right)}^{\ast}\right] $$

In this equation, ${\mathrm{S}}_{\mathrm{ij}}^{\mathrm{OF}}$ is the second-order transition moment given by:

7 $$ {S}_{ij}^{OF}\left({\zeta}_1,{\zeta}_2\right)=\frac{1}{\mathrm{\hslash}}\sum \limits_K\left[\frac{\left\langle 0\left|{\zeta}_1\cdot {\overset{\frown }{\mu}}_i\right|K\right\rangle \left\langle K\left|{\zeta}_2\cdot {\overset{\frown }{\mu}}_j\right|F\right\rangle }{\omega_{\alpha }-{\omega}_1}+\frac{\left\langle 0\left|{\zeta}_2\cdot {\overset{\frown }{\mu}}_i\right|K\right\rangle \left\langle K\left|{\zeta}_1\cdot {\overset{\frown }{\mu}}_j\right|F\right\rangle }{\omega_{\alpha }-{\omega}_2}\right] $$

where ℏ*ω*_1_ + ℏ*ω*_2_ should satisfy the resonance condition and $\left\langle 0\left|{\zeta}_1\cdot {\overset{\frown }{\mu}}_i\right|K\right\rangle$ stands for the transition moment between electronic states |0> and |K>, respectively. *ζ* is the vector defining polarization of photons. To describe the two-photon allowed states, the quadratic response functions formalism [49, 50] within the DFT framework was used as implemented in the DALTON 2011 program [51, 52]. Solvent effects were taken into account with the self-consistent reaction field (SCRF) model. All the 2PA calculations were carried out employing the CAM-B3LYP functional and the 6-311++G(d,p) basis set.

The biological activities were simulated using a combination of the 3D/4D QSAR BiS/MC and CoCon algorithms [53–55].

The binding properties of considered dyes were studied by performing series of AutoDock 4.2 [56–58] and AutoDock Vina simulations. For each active complex comprising Concanavalin A (ConA), their crystal structures were taken from PDB ID: 2a7a [59]. The cubic grid box with the dimensions of 16 Å and a grid spacing of 1 Å was set up in such way that the reactive -NH2 groups of Lysine were at its center. In order to identify appropriate binding energy and conformation of compounds, the Lamarkian genetic algorithm was employed. For each atom of the receptor molecule, Gasteiger charges were calculated. The investigation of the binding site was performed using a united-atom scoring function. For each amino acid, the docking simulations were performed tenfold.

## Results

### Physicochemical properties

For all tested derivatives, the charge-transfer (CT) excitation corresponds to the HOMO→LUMO transition. In the case of NH_2_ molecule, energy separation between HOMO-LUMO orbital (E_GAP_, Table S1) slightly decreases with increasing solvent polarity. However, for the NH_2_ derivative, ${\Delta  E}_{GAP}^{THF- Water}$ = 0.03 eV. Changing the substituent also has little effect on these values. Only conversion of the -NH_2_ substituent to -NO_2_ reduces E_GAP_ and for water ${\Delta  E}_{GAP}^{NH_2-{NO}_2}$ = 0.0613 eV. After conjugation with the protein, the E_GAP_ value increases on average by 0.6 eV. Only for the -NO_2_ substituent it is reduced by 0.3 eV. In addition, for most of the conjugates, E_GAP_ also decreases with an increase of the medium polarity; for NO_2_Con this value increases. In addition, after the chemical hardness (*η*) analysis, the tested derivatives should be treated as soft molecules. A high electronegativity (*χ*) value suggests an easy formation of covalent bonds during various chemical processes.

**Fig. 2:**
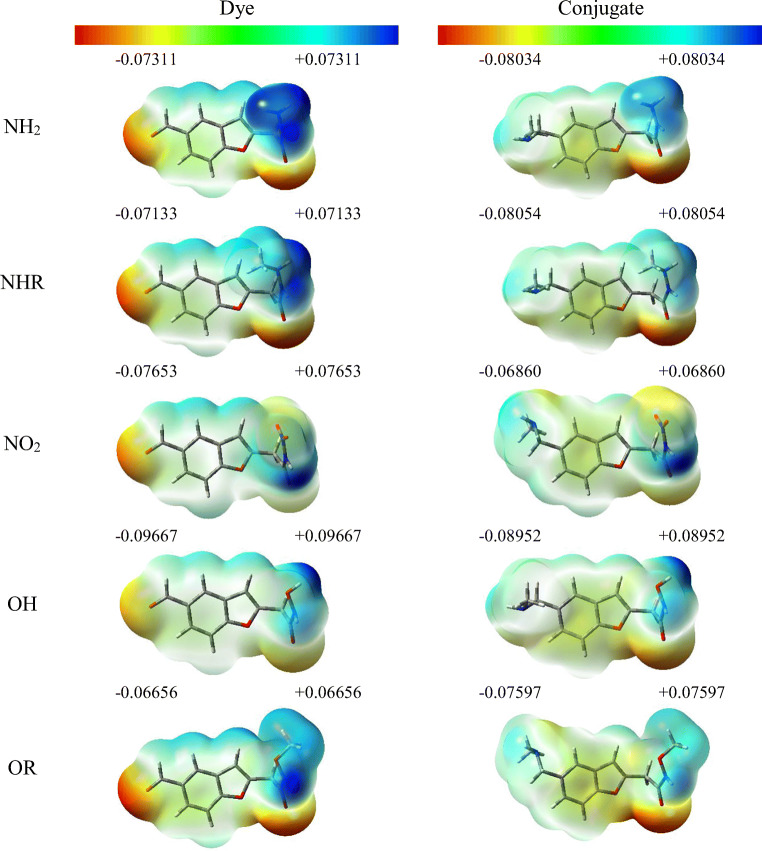
The MEP surfaces for benzofuran derivatives in water. Values are given in [a.u]

For the assessment of the sites of potential electrophilic and nucleophilic attack, the molecular electrostatic potential (MEP) analysis was performed, as seen in Fig. 2. For the NH_2_ derivative, it is the nitrogen atom with hydrogen attached to electron-donating group that is characterized with the highest electronegativity and it is therefore the most potent for a nucleophilic attack (positive, blue zones). Different substituents do not alter the location of these sites. On the other hand, the sites for electrophilic attack (negative, red, and yellow zones) are located mainly on the aldehyde group and the oxygen atom connected by a double bond with the carbon atom with attached NH_2_ group. As it was in the former case, changing the substituent does not result in changing of the location of these sites. When taking into account all the studied molecules, it is the OH derivative that can undergo an electrophilic attack most readily as the charge on the oxygen atom is as high as − 0.09667 a.u. After conjugation with ConA, places for a potential nucleophilic attack do not change. However, the only site for an electrophilic attack is an oxygen atom near -NH2, while the zone at the marker-protein link disappears.

**Fig. 3:**
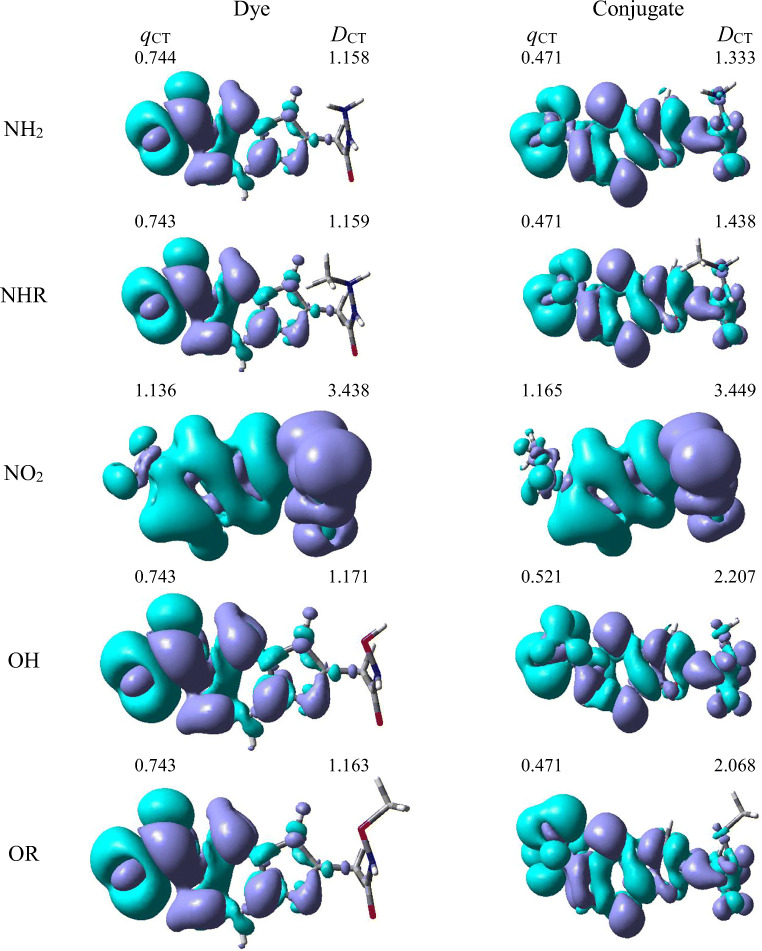
Density difference plot in water. The purple and green lobes correspond to (*ρ*_*−*_) and (*ρ*_*+*_), respectively

The studies have been devoted to many reflections on spectral properties, corresponding to the HOMO→LUMO photoexcitation (π-π^*^ transitions). In order to estimate contributions from other orbitals and determine the nature of electronic states, the density variation upon photoexcitation (Δ*ρ*(r)) was computed for the first electronic transitions, which is graphically depicted in Fig. 3. For BIN-T, the figure indicates that the depletion zones (blue) are mainly located on aldehyde group, while in contrast, growth zones (purple) on benzofuran part. The presence and change of donor-acceptor substituents does not change the position of these zones. The exception is the NO_2_ derivative, for which depletion zones include not only the aldehyde group but also benzofuran part. In contrast, purple zones extend mainly to parts with an electron-withdrawing substituent. Protein conjugation does not significantly change the position of these zones.

On the other hand, the polarity of the environment does not significantly affect the parameters describing Δ*ρ*(r) (Table S3). For all derivatives, the amount of the transferred charge is not variable as a function of solvent polarity and is at 0.743–0.744 a.u. level. As for previous quantities, the exception is the NO2 marker, for which *q*_CT_ is 1.136 a.u. The polarity of the environment also significantly affects the charge-transfer distance and increases it from 1.118 Å in THF to 1.158 Å in water for NH_2_ molecule. For other derivatives, the *D*_CT_ value is at a similar level and for example a difference ${\Delta  D}_{CT}^{OR-{NH}_2}$ is only 0.005 Å. However, for the NO_2_ derivative, the *D*_CT_ value is three times higher than the other molecules and exceeds 3.4 Å. Conjugation reduces the *q*_CT_ value by almost half and for NH_2_ in water ${\Delta  q}_{CT}^{NH_2-{NH}_2 Con}$ is 0.273 a.u. (Tab. S4). The exception is again NO_2_, for which these values remain practically unchanged. In addition, *D*_CT_ increases after conjugation with the protein. While for NH_2_ ${\Delta  D}_{CT}^{NH_2 Con-{NH}_2}$ is only 0.175 Å, it increases to 1.036 Å for the OH derivative. Solvent effects on Δ*ρ*(r) parameters remain the same as for markers before combining with the macromolecule. The presented analysis indicates the charge-transfer nature of tested derivatives. It also confirms the contributions from the HOMO-LUMO transition. At the same time, it indicates the possibility of the presence of contributions from other orbitals.

All tested markers are characterized by good solubility in all discussed media (Tab. S). Δ*G*_*solv*_ increases with the increase in the polarity of the environment. Only the transition from DMSO to water creates a slight decrease and for NH_2_ ΔΔ*G*_*sol*_ is 1.51 kcal/mol. At the same time, Δ*G*_*sol*_ in water is slightly lower than the values in THF and ΔΔ*G*_*sol*_ is 0.55 kcal/mol. After conjugation, the solubility practically does not change, and the Δ*G*_*sol*_ value increases slightly (Tab. S6). For the reference NH_2_, ΔΔ*G*_*sol*_ in THF is 0.07 kcal/mol and increases to 0.47 kcal/mol in water. Identical relationships are observed for the remaining markers.

### Linear optical properties

Theoretical linear optical properties of analyzed markers are shown in Tables 1 and SI7. Due to the high compatibility of theoretical and experimental values obtained on the basis of PBE0 [33–36], the spectroscopic parameters were determined using this function. It also accurately predicts NH_2_ values recorded experimentally. For vertical values, the ${\Delta \lambda}_{ABS}^{TDDFT- EXP}$difference in THF is 23.46 nm but in DMF it is only 1.10 nm. For the values obtained within the cLR approximation, this error increases to the levels of 25.46 nm and 5.83 nm, respectively. In the case of benzofuran derivatives, the presence of donor-acceptor substituents results in week shift in absorption maximum (*λ*_ABS_). Attaching a methyl group to the -NH_2_ results in a batochromic shift. However, the magnitude of this shift is insignificant and for vertical values is only 0.06 nm on average. In turn, the replacement of the amino substituent with -OH also shifts *λ*_ABS_ in the direction of longer wavelengths by 0.20 nm on average. Attaching an additional -CH_3_ group to the hydroxyl substituent results in a hypsochromic shift and Δ*λ*_ABS_ is on average 0.07 nm. For the investigated benzofuran derivatives, the largest solvatochromic shift is observed in MeCN and the smallest in DMS. Nevertheless, conversion of -NH_2_ with -NHCH_3_, -OH, and -OCH_3_ substituents does not cause significant changes in the location of the maximum absorption in all analyzed solvents. A significant shift is obtained when substituting the amino group with a -NO_2_ group. The average value of the batochromic shift ${\Delta \lambda}_{ABS}^{NO_2-{NH}_2}$ is at the level of 30.30 nm. Identical conclusions can be drawn after analyzing the maintained values within the cLR approximation. Conjugation with ConA is followed by another solvatochromic shift. In the case of NH_2_, NHR, OH, and OR derivatives, a shift towards shorter waves is observed. The size of this slide is significant and amounts to an average of 54 nm. In turn, there is a batchromic shift for the NO_2_ probe, however its size is smaller compared with other probes and on average is 27 nm.

**Table 1:** Linear and nonlinear optical properties in water

	${\lambda}_{ABS}^{TDDFT}$	*f*	${\lambda}_{ABS}^{cLR}$	μ_GS_	μ_CT_	${\lambda}_{FL}^{TDDFT}$	〈*α*〉	Δ*α*	*β*_*vec*_	〈*δ*^*OF*^〉	${\sigma}_{OF}^{(2)}$
NH_2_	313.94	1.1132	317.10	9.50	16.31	396.32	212.90	96.33	669.93	0.06	0.00
NHR	313.98	0.7062	317.12	9.43	16.89	396.33	228.99	92.95	610.60	0.06	0.00
NO_2_	314.12	1.0190	317.28	5.26	15.44	434.56	223.63	82.90	526.91	626.96	2.18
OH	314.09	1.0666	317.23	9.28	17.69	396.67	207.11	102.87	713.63	0.05	0.00
OR	344.42	1.0461	338.14	9.72	18.12	396.64	223.56	96.77	639.03	0.06	0.00
NH_2_Con	259.34	1.2824	258.88	8.01	13.91	356.88	233.11	92.48	0.75	18.05	0.08
NHRCon	259.44	1.2661	259.14	8.08	13.91	356.89	249.23	76.22	1.02	17.65	0.08
NO_2_Con	260.39	1.2749	260.64	2.43	21.68	412.67	244.02	72.19	3.31	1204.90	3.82
OHCon	259.90	1.1615	260.37	6.86	12.70	357.23	227.49	99.17	0.12	32.85	0.15
ORCon	371.08	1.0435	365.39	5.58	11.02	357.20	243.36	90.25	1.88	32.85	0.15

The position of maximum absorption is slightly sensitive to environmental changes, for both initial markers and conjugates. The *λ*_ABS_ value monotonously decreases as the polarity of the medium increases. However, the transition from weakly polar THF to water is accompanied by an increase in excitation energy of just 1.5 nm. Also, ${\Delta \lambda}_{ABS}^{marker- conjugate}$ decreases with increasing solvent polarity.

The interconnectedness of the analysis of spectroscopic parameters with MEP indicates the possibility of specific interactions in the solute-solvent system. A monotonic increase in excitation energy indicates on larger polarization and better S_g_ stabilization. However, this is not consistent with the polarity of the excited state (Δ*μ*_*CT* − *g*_). Hypsochromic shift, as the effect of the increase in environmental polarity, should result in the μ_g_ > μ_CT_ relationship. In contrast, for both markers and conjugates, there is an inverse relationship (Tables S11 and S12), which is a characteristic of positive solvatochromism. In any case, the polarity of the excited state also changes monotonously. In all media, the highest Δ*μ*_*CT* − *g*_ value is a characteristic for NO_2_ marker and in terms of average values probes can be ranked as follows: NO_2_ (Δ*μ*_*CT* − *g*_ = 10.98 D) > OH = OR (Δ*μ*_*CT* − *g*_ = 8.30 D) > NH_2_ = NHR (Δ*μ*_*CT* − *g*_ = 7.20 D). In addition, as the polarity of the medium increases, the dipole moment values increase and both ${\mu}_g^{Water- THF}$ and ${\mu}_{CT}^{Water- THF}$ do not exceed 0.8 D. In terms of μ_g_ value, the molecules can be ordered as follows: OR > NH_2_ = NHR > OH > NO_2_, and for μ_CT_: OR > OH > NHR > NH_2_ > NO_2_. The polarity of the excited state decreases after conjugation. The smallest differences in ΔΔ*μ*_*CT* − *g*_ are visible for the NH_2_ derivative (1.27 D), and the largest for OR (2.61 D). Only for the NO_2_ probe, an increase in CT state polarity is observed, by 8.98 D on average. This is due to changes in dipole moment values. In all cases, μ_g_ and μ_CT_ decreases. Only for NO_2_, μ_CT_ increases by an average of 6.27 D.

Tables 1 and S13 show the values of the de-excitation energy (*λ*_FL_). Also, in this case the functional PBE0 perfectly reproduces the values and ${\Delta \lambda}_{FL}^{TDDFT- Exp}$ is only 3.35 nm. As for *λ*_ABS_, the location of maximum fluorescence for NH_2_, NHR, OH, and OR derivatives lies in close proximity. The OH spectrum is the most shifted towards longer wavelengths, while NH_2_ is the most shifted towards shorter wavelengths. However, the ${\Delta \lambda}_{FL}^{OH-{NH}_2}$ difference is only 0.34 nm. The most batochromic shifted spectrum is NO_2_ and ${\Delta \lambda}_{FL}^{NO_2-{NH}_2}$ is 40.98 nm. Also in this case, the energy of de-excitation increases monotonously as a function of the polarity of the medium. However, again these differences are insignificant and ${\Delta \lambda}_{FL}^{THF- Water}$ for NH_2_ is 5.30 nm. The largest solvatochromic shift is observed for the NO2 probe, where ${\Delta \lambda}_{FL}^{THF- Water}$ = 12.13 nm. Conjugation promotes an additional increase in de-excitation energy. The size of the hypsochromic shift is the smallest for NO_2_ and is on average 19.70 nm. For other probes, it is at the same level and amounts to 39.30 nm.

All tested derivatives have a high Stokes shift value (Δ*ν*^*St*^), in the range 6500–6800 cm^−1^. In each case, its value decreases monotonously as a function of the solvent polarity. However, the transition from THF to water decreases the Δ*ν*^*St*^ value by only 180 cm^−1^. Only for NO_2_, this decrease is greater and ΔΔ*ν*^*St*^ is 571.88 cm^−1^. In terms of this quantity, the analyzed probes can be ranked as follows: NO_2_ < NHR < NH_2_ < OH < OR, with ${\Delta \nu}_{OR- NHR}^{St}$ = 11.01 cm^−1^. After conjugation with ConA, the Δ*ν*^*St*^ value for NO_2_ decreases by 3186.65 cm^−1^ on average. For the remaining probes, this quantity increases, and the largest increase is observed for NH_2_ (3951.81 cm^−1^). Although all derivatives are described by a high Δ*ν*^*St*^ value, the presence of -NO_2_ will be the least desirable substituent in the marker structure. However, taking into account all linear optical parameters, it allows obtaining a probe with a different spectrum of operation.

### Nonlinear optical properties

The polarizability (〈*α*〉) and first hyperpolarizability (*β*_vec_) of molecules irradiated with intense laser light giving the electric field is the subject of many research in terms of understanding various nonlinear optical properties (NLO). In particular, these studies include the interrelationship of NLO with the electronic structure to design new multifunctional fluorescence probes. The calculated values of 〈*α*〉 and *β*_vec_ are collected in Tables 1, S15, and S16. In the case of 〈*α*〉, the analyzed compounds can be ordered in the following way: 〈*α*〉^*OH*^ < ${\left\langle \alpha \right\rangle}^{NH_2}$ < 〈*α*〉^*OR*^ < ${\left\langle \alpha \right\rangle}^{NO_2}$ < 〈*α*〉^*NHR*^. With the increase in the solvent polarity, the polarizability value increases and the transition from THF to water is accompanied by an average increase of 12 a.u. At the same time, the polarity of the medium does not affect the alignment of the studied molecules in terms of 〈*α*〉. After the reaction with ConA, the polarizability value increases on average by 19–21 a.u. Like the solvent power, conjugation does not affect the ranking of the tested derivatives. For the first hyperpolarizability, benzofuran derivatives can be ordered inversely to 〈*α*〉: ${\beta}_{vec}^{NO_2}$ < ${\beta}_{vec}^{NHR}$ < ${\beta}_{vec}^{OR}$ < ${\beta}_{vec}^{NH_2}$< ${\beta}_{vec}^{OH}$. Also in this case, the *β*_vec_ value increases monotonously as the medium permeability increases, and the transition from THF to water increases in the range of 111 a.u. up to 127 a.u. More importantly, for each derivative after conjugation *β*_vec_ drops below 4 a.u. Since the nonlinear response will be derived from the structure obtained after conjugation with protein, markers based on the benzofuran backbone are not suitable for NLO processes. At the same time, in this case the power of the donor/acceptor substituents does not affect the intensification of the nonlinear response, and thus it cannot be efficient e.g. in second-harmonic generation (SHG).

Table S1 shows the value of two-photon absorption cross section (TPA, 〈*δ*^*OF*^〉). In general, it is difficult to decisively conclude about the effect of the solvent on the 〈*δ*^*OF*^〉 value. For derivatives before the reaction with ConA, these values are practically zero and only for NO_2_ in water a slight increase is observed. However, 〈*δ*^*OF*^〉 is still very small and is only 626 a.u. Conjugation becomes a factor that maximizes the value of TPA. However, in all media, 〈*δ*^*OF*^〉 does not exceed the value of 33 a.u. Again, the largest increase is observed for the NO_2_ derivative, because for this molecule a larger increase is visible and D〈*δ*^*OF*^〉 reaches the value of 1204.90 in water. In this case, the TPA value also increases monotonously as a function of solvent polarity. However, in order to compare the calculated values of the TPA with those determined experimentally (${\sigma}_{OF}^{(2)}$), the relation 5 was used. In this equation, the broadening of the final state due to its finite lifetime 0.25 eV was assumed. The effect of the solvent on the ${\sigma}_{OF}^{(2)}$ value remains the same as for 〈*δ*^*OF*^〉. For all molecules, except NO_2_, ${\sigma}_{OF}^{(2)}$ is 0 GM. For this compound, only in water the value is greater than 0 GM and is 2.18 GM. Conjugation is not conducive to improving these values. Only for NO_2_Con the ${\sigma}_{OF}^{(2)}$ increase is observed; however, it is small and in THF is only 3.65 GM and in water 1.64 GM. Based on this analysis, to obtain a probe with a high TPA value, used as tools in real-time dynamic in vivo and in vitro research, an additional chromophore group intensifying the NLO response should be added to the structure of the analyzed dyes. In particular, it will be desirable to modify the derivative containing the -NO_2_ substituent.

### Biological activities

**Fig. 4:**
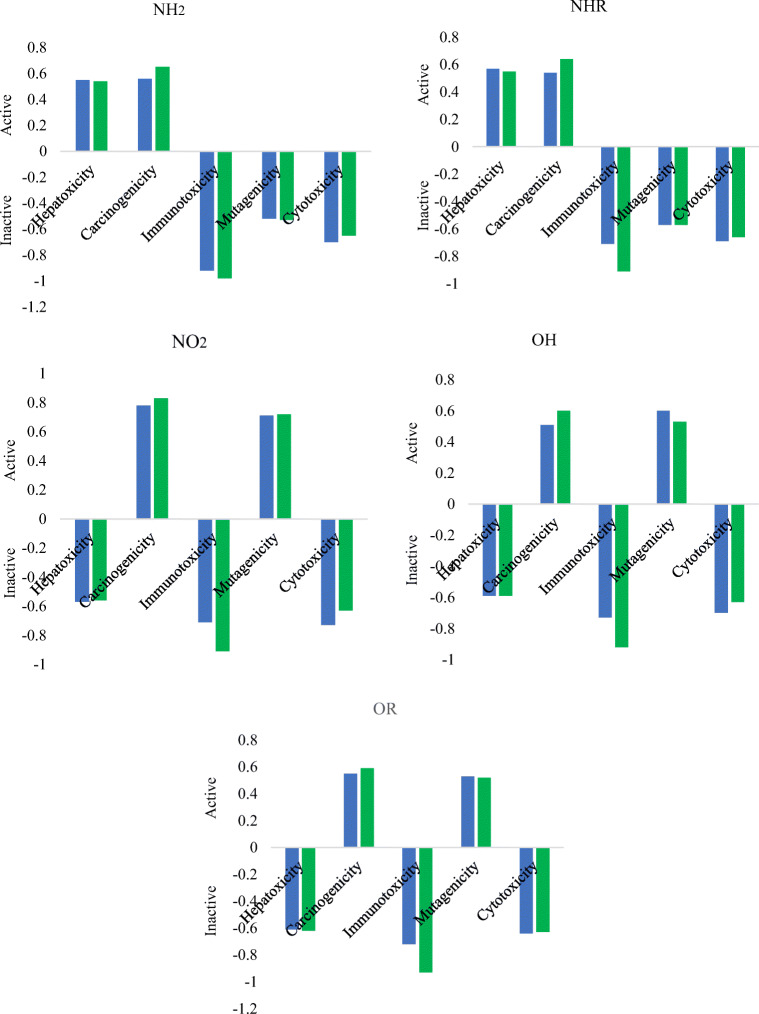
Theoretically determined toxicological parameters. Blue line, markers before conjugation; green line, conjugates

The tested derivatives are described by relatively good bioavailabilty (LogP > 2.5). This suggests good permeability through cell membranes and achieving adequate concentration at the site of interaction with proteins. After conjugation, this value exceeds 3.5. Only for OHCon and ORCon it drops to 1.08 and 1.80, respectively. A calculated LogBCF value in the range of 2.1–2.3 indicates the lack of bioaccumulation in the tissues of living organisms and the ease of excretion with urine. At the same time, the reaction with ConA does not affect the value of LogBCF. For this reason, all derivatives should not bioaccumulate after fulfilling their optical role. Moreover, the tested molecules before and after conjugation are characterized by high metabolism by CYP450-2D6 and Cyp450-3A4 (Tables S17 and S18). The above observations indicate the fact that both forms of markers will be promptly removed from the tissues and will not interact with different drugs or biomolecules. The oral toxicity value calculated as LD_50_ > 1800 mg/kg classifies the studied derivatives in class 4 of the degree of toxicity which means that they can be regarded as essentially nontoxic for humans. The toxicity of the studied molecules is not influenced by the presence of different substituents; however, they do alter their other toxicological parameters (Fig. 4). None of the derivatives shows immunotoxicity and cytotoxicity. However, all derivatives exhibit carcinogenicity properties. The presence of the -NH_2_ and -NHCH_3_ substituents causes hepatoxicity but no mutagenicity. Other derivatives are characterized by inverse relationships. In addition, conjugation does not affect the reported toxicity properties. On the other hand, all molecules, before and after attachment to a macromolecule, have other biological activities suggesting their potential use in other areas of medicine. All derivatives, except NO_2_, are characterized by high antibacterial activity (*P* > 70%). However, only the NO_2_ derivative has antioxidant properties (*P* > 57%). Conjugation with ConA causes antioxidant properties to occur in all molecules, and there is no change in the antibacterial property. In addition, the tested benzofuran derivatives are characterized by many other activities, such as alpha-radioprotector activity, acyl-CoA-holesterol transferase inhibitory activity, gamma-radioprotector activity mechanism I, antiadenovirus activity, antipsychotic activity diazepine site, antitumor alkylic activity, antitumor DNA antimetabolitic activity, antitumor topoisomerase I inhibitory activity, and HIV1-proteaze inhibitory activity. At the same time, the influence of subsequent substituents on the maximization or reduction of these activities is demonstrated in Tables S17 and S18.

### AutoDock simulations

**Fig. 5:**
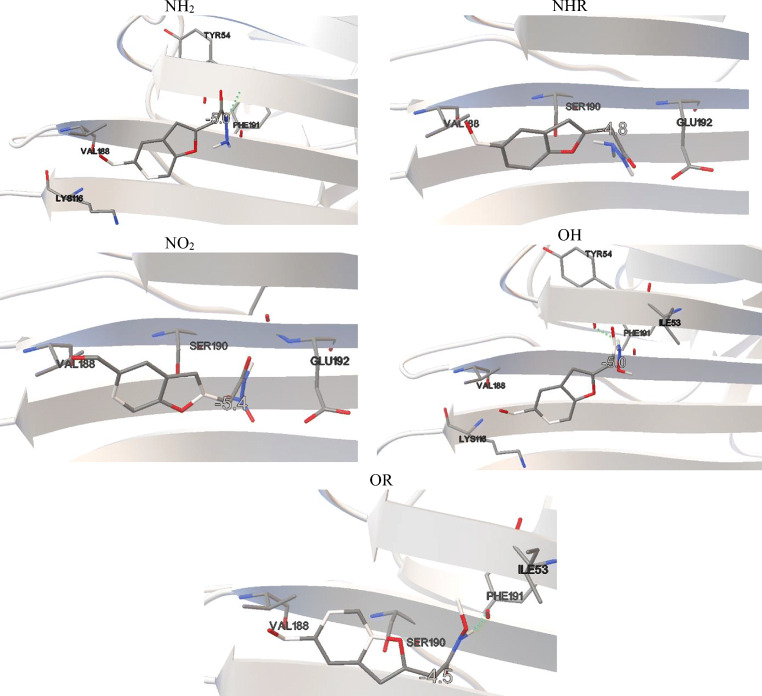
Results of the AutoDock simulations

As it was stated before, the considered benzofuran derivatives cannot be used in the studies on two-photon absorption. They can be utilized however as fluorescent markers in single-photon studies, which makes them suitable for application as alternative markers in fluorescent probes used for medical imaging. It is the reaction with lysine through which the conjugation with the protein occurs. For this particular study, Concanavalin A was chosen and the conjugation to ConA molecule occurs via LYS114 (Fig. 5). The presence of different substituents does not change the active binding site of the studied derivatives to the protein molecule. Among studied derivatives, the highest affinity for this active center is shown by the NO2 derivative. In this case, the binding energy (*ΔG*_*b*_) was calculated to be − 5.4 kcal/mol (Table S19), while the inhibition constant (*K*_*i*_) of this binding site was found to be 1.54 μM. An aromatic cage formed by LYS114, VAL188, SER190, and GLU192 is the site for the insertion of the molecule. No π-π* interactions were found the active site, neither it is stabilized by H-bonds. Other two derivatives, namely NH_2_ and OH, are characterized by a slightly lower affinity for ConA with *ΔG*_*b*_ = − 5.0 kcal/mol and the corresponding inhibition constants equal 2.19 μM and 2.47 μM, respectively.

The active impact cavity here is LYS116, VAL188, TYR54, and PHE191. In the case of OH, ILE53 also interacts here. In this case, hydrogen bonds are observed but no π-π* interactions. For the first molecule, the H-bond is formed between the hydrogen atom of the first NH group at the donor substituent and the nitrogen atom TYR54. In the second case, the same hydrogen will interact with the oxygen atom TYR54. OR has the lowest affinity for the protein, for which Δ*G*_*b*_ = − 4.5 kcal/mol and *K*_*i*_ = 5.14 μM. During conjugation, the C=O bond raptures and then a new one forms with the -NH2 group of protein. The rate of biocomplex creation will therefore also be affected by the energy barrier (Δ*E*) necessary to overcome. The lowest Δ*E* value when moving the oxygen atom away from the aldehyde group at a distance of 2.3 Å is observed for NO_2_ derivative and is 20.22 kcal/mol. A slightly higher value is observed for NH_2_ and NHr for which Δ*E* is 22.13 kcal/mol and 22.14 kcal/mol, respectively. The strongest bond, and thus hindering the conjugation, is the C=O in OH and OR, where Δ*E* is 23.16 kcal/mol and 23.26 kcal/mol, respectively. In addition, no significant structural changes are observed for any molecule due to matching with the aromatic cavity.

## Conclusions

This paper presents the effect of modulation of benzofuran derivatives structure to optimize linear and nonlinear optical properties and biological activities. The obtained results were compared with those obtained for probes after conjugation with Concanavalin A. Regardless of the type of substituent used, the CT excitation corresponds to the HOMO-LUMO transition. Analysis of optical properties showed that the -NHCH_3_, -OH, and -OCH_3_ substituents do not induce significant batochromic shifts of maximum absorption and fluorescence relative to the reference -NH_2_. They also do not affect dipole moment values and nonlinear optical properties. Only substitution of the amino group with the -NO_2_ significantly affects the batochromic shift, maximum absorption, and fluorescence, as well as nonlinear optical properties. The presence of the -NO_2_ group also enhances the polarity of the CT state. All derivatives exhibit a lack of bioaccumulation in the tissues of living organisms and an easy excretion with urine after fulfilling their role as a fluorescent marker. The substitution of substituents does not affect the LD_50_, which high value suggests no toxicity to living organisms. On the other hand, however, change of the substituent can induce hepatoxicity (NH_2_ and NHR) and mutagenicity (NO_2_, OH, OR). All substituents direct the probe to the same active center (LYS114) through which the biocomplex is formed. None of the tested markers are suitable for use in two-photon studies. In addition, conjugation promotes hypsochromic shift in the position of maximum absorption and fluorescence for all analyzed markers. The smallest shift is observed for the NO_2_ derivative. Also, for this substituent, conjugation slightly improves the two-photon absorption cross section value. In summary, all analyzed fluorescent probes are suitable for medical imaging applications. Nevertheless, the most valuable alternative is the NO_2_ derivative. Expanding its structure with additional substituents will allow obtaining valuable probes with the desired properties for use in in vivo and in vitro bioimaging.

## Electronic supplementary material

ESM 1 Linear and nonlinear optical properties and biological activities for investigated compounds (DOCX 86 kb)

## Abbreviations

XCon: Marker determination after conjugation
ConA: Concanavalin A
THF: Tetrahydrofuran
MeAc: Acetone
MeCN: Acetonitrile
DMF: n,n-Dimethylformamide
DMSO: Dimethylsulfoxide
