# Supplementary material for: Modulation of benzofuran structure as a fluorescent probe to optimize linear and nonlinear optical properties and biological activities
Source: J Mol Model. 2020 Sep 19;26(10):272. doi: 10.1007/s00894-020-04539-6 (PMC7502069; doi:10.1007/s00894-020-04539-6)
Supplement: Supplementary file 1 — Linear and nonlinear optical properties and biological activities for investigated compounds (DOCX 86 kb) [file 894_2020_4539_MOESM1_ESM.docx]

**Modulation of benzofuran structure as a fluorescent probe to optimize linear and nonlinear optical properties and biological activities**

Przemysław Krawczyk^1*^

^1^Nicolaus Copernicus University, Collegium Medicum, Faculty of Pharmacy, Department of Physical Chemistry, Kurpińskiego 5, 85-950 Bydgoszcz, Poland

*Corresponding author: przemekk@cm.umk.pl; tel: +48 52 5853679

**Supporting Informtion**

| Table |  | Page |
| --- | --- | --- |
| 1 | The frontier orbital energies in selected solvents for benzofuran derivatives. All values are given in eV | 2 |
| 2 | The frontier orbital energies in selected solvents for coniugates. All values are given in eV | 3 |
| 3 | CT parameters for the bright low-lying excited state for benzofuran derivatives | 4 |
| 4 | CT parameters for the bright low-lying excited state for conjugates | 4 |
| 5 | Free energies (Δ*G*_solv_, kcal/mol) of solvation for benzofuran derivatives | 4 |
| 6 | Free energies (Δ*G*_solv_, kcal/mol) of solvation for conjugates | 4 |
| 7 | The vertical excitation energies (in nm) for benzofuran derivatives | 4 |
| 8 | The cLR corrected excitation energies (in nm) for benzofuran derivatives | 5 |
| 9 | The vertical excitation energies (in nm) for conjugates | 5 |
| 10 | The cLR corrected excitation energies (in nm) for conjugates | 5 |
| 11 | Calculated values of dipole moments (in D) for the ground and CT excited state fo benzofuran derivatives | 5 |
| 12 | Calculated values of dipole moments (in D) for the ground and CT excited state for conjugates | 6 |
| 13 | The vertical de-excitation energies (in nm) for benzofuran derivatives | 6 |
| 14 | The vertical de-excitation energies (in nm) for conjugates | 6 |
| 15 | Nonlinear optical properties for benzofuran derivatives. Values are given in a.u. | 7 |
| 16 | Nonlinear optical properties for conjugates. Values are given in a.u. | 7 |
| 17 | Biological activities for benzofuran derivatives | 8 |
| 18 | Biological activities for conjugates | 9 |
| 19 | Binding free energies (Δ*G_b_*, kcal/mol) obtained during AutoDock simulations | 10 |

Table S1. The frontier orbital energies in selected solvents for benzofuran derivatives. All values are given in eV

| NH2 | THF | MeAC | MeCN | DMF | DMSO | Water |
| --- | --- | --- | --- | --- | --- | --- |
| E_HOMO_ | -6.7058 | -6.7123 | -6.7142 | -6.7142 | -6.7147 | -6.7156 |
| E_LUMO_ | -1.7923 | -1.8195 | -1.8269 | -1.8271 | -1.8290 | -1.8320 |
| E_GAP_ | 4.9135 | 4.8928 | 4.8873 | 4.8871 | 4.8857 | 4.8835 |
| *ƞ* | 2.4567 | 2.4464 | 2.4437 | 2.4435 | 2.4429 | 2.4418 |
| *μ* | -4.2490 | -4.2659 | -4.2705 | -4.2707 | -4.2719 | -4.2738 |
| *χ* | 4.2490 | 4.2659 | 4.2705 | 4.2707 | 4.2719 | 4.2738 |
| NHR |  |  |  |  |  |  |
| E_HOMO_ | -6.7123 | -6.7186 | -6.7202 | -6.7205 | -6.7210 | -6.7218 |
| E_LUMO_ | -1.7931 | -1.8198 | -1.8269 | -1.8274 | -1.8293 | -1.8323 |
| E_GAP_ | 4.9192 | 4.8988 | 4.8933 | 4.8930 | 4.8917 | 4.8895 |
| *ƞ* | 2.4596 | 2.4494 | 2.4467 | 2.4465 | 2.4458 | 2.4448 |
| *μ* | -4.2527 | -4.2692 | -4.2735 | -4.2739 | -4.2752 | -4.2771 |
| *χ* | 4.2527 | 4.2692 | 4.2735 | 4.2739 | 4.2752 | 4.2771 |
| NO2 |  |  |  |  |  |  |
| E_HOMO_ | -6.9420 | -6.9221 | -6.9172 | -6.9169 | -6.9156 | -6.9134 |
| E_LUMO_ | -2.5192 | -2.4963 | -2.4903 | -2.4901 | -2.4887 | -2.4863 |
| E_GAP_ | 4.4228 | 4.4258 | 4.4269 | 4.4269 | 4.4269 | 4.4271 |
| *ƞ* | 2.2114 | 2.2129 | 2.2134 | 2.2134 | 2.2134 | 2.2136 |
| *μ* | -4.7306 | -4.7092 | -4.7038 | -4.7035 | -4.7021 | -4.6998 |
| *χ* | 4.7306 | 4.7092 | 4.7038 | 4.7035 | 4.7021 | 4.6998 |
| OH |  |  |  |  |  |  |
| E_HOMO_ | -6.7613 | -6.7792 | -6.7814 | -6.7814 | 6.7820 | -6.7828 |
| E_LUMO_ | -1.7975 | -1.8285 | -1.8361 | -1.8367 | -1.8388 | -1.8421 |
| E_GAP_ | 4.9638 | 4.9507 | 4.9453 | 4.9448 | -8.6208 | 4.9407 |
| *ƞ* | 2.4819 | 2.4754 | 2.4726 | 2.4724 | -4.3104 | 2.4703 |
| *μ* | -4.2794 | -4.3039 | -4.3088 | -4.3090 | 2.4716 | -4.3124 |
| *χ* | 4.2794 | 4.3039 | 4.3088 | 4.3090 | -2.4716 | 4.3124 |
| OR |  |  |  |  |  |  |
| E_HOMO_ | -6.7613 | -6.7724 | -6.7814 | -6.7754 | -6.7760 | -6.7828 |
| E_LUMO_ | -1.7975 | -1.8258 | -1.8361 | -1.8342 | -1.8367 | -1.8421 |
| E_GAP_ | 4.9638 | 4.9467 | 4.9453 | 4.9412 | 4.9393 | 4.9407 |
| *ƞ* | 2.4819 | 2.4733 | 2.4726 | 2.4706 | 2.4697 | 2.4703 |
| *μ* | -4.2794 | -4.2991 | -4.3088 | -4.3048 | -4.3063 | -4.3124 |
| *χ* | 4.2794 | 4.2991 | 4.3088 | 4.3048 | 4.3063 | 4.3124 |

Table S2. The frontier orbital energies in selected solvents for coniugates. All values are given in eV

| NH_2_Con | THF | MeAC | MeCN | DMF | DMSO | Water |
| --- | --- | --- | --- | --- | --- | --- |
| E_HOMO_ | -6,3305 | -6,3659 | -6,3724 | -6,3727 | -6,3754 | -6,3795 |
| E_LUMO_ | -0,7715 | -0,8077 | -0,8161 | -0,8167 | -0,8194 | -0,8240 |
| E_GAP_ | 5,5590 | 5,5582 | 5,5563 | 5,5560 | 5,5560 | 5,5554 |
| *ƞ* | 2,7795 | 2,7791 | 2,7781 | 2,7780 | 2,7780 | 2,7777 |
| *μ* | -3,5510 | -3,5868 | -3,5943 | -3,5947 | -3,5974 | -3,6018 |
| *χ* | 3,5510 | 3,5868 | 3,5943 | 3,5947 | 3,5974 | 3,6018 |
| NHRCon |  |  |  |  |  |  |
| E_HOMO_ | -6,3367 | -6,3697 | -6,3792 | -6,3797 | -6,3825 | -6,3868 |
| E_LUMO_ | -0,7737 | -0,8107 | -0,8208 | -0,8213 | -0,8240 | -0,8281 |
| E_GAP_ | 5,5631 | 5,5590 | 5,5584 | 5,5584 | 5,5584 | 5,5587 |
| *ƞ* | 2,7815 | 2,7795 | 2,7792 | 2,7792 | 2,7792 | 2,7793 |
| *μ* | -3,5552 | -3,5902 | -3,6000 | -3,6005 | -3,6032 | -3,6075 |
| *χ* | 3,5552 | 3,5902 | 3,6000 | 3,6005 | 3,6032 | 3,6075 |
| NO_2_Con |  |  |  |  |  |  |
| E_HOMO_ | -6,5779 | -6,5920 | -6,5964 | -6,5966 | -6,5980 | -6,6004 |
| E_LUMO_ | -2,4479 | -2,4525 | -2,4533 | -2,4533 | -2,4536 | -2,4541 |
| E_GAP_ | 4,1300 | 4,1395 | 4,1430 | 4,1433 | 4,1444 | 4,1463 |
| *ƞ* | 2,0650 | 2,0697 | 2,0715 | 2,0717 | 2,0722 | 2,0732 |
| *μ* | -4,5129 | -4,5223 | -4,5248 | -4,5250 | -4,5258 | -4,5273 |
| *χ* | 4,5129 | 4,5223 | 4,5248 | 4,5250 | 4,5258 | 4,5273 |
| OHCon |  |  |  |  |  |  |
| E_HOMO_ | -6,3762 | -6,4124 | -6,3762 | -6,4227 | -6,4255 | -6,4301 |
| E_LUMO_ | -0,8559 | -0,8757 | -0,8559 | -0,8844 | -0,8869 | -0,8907 |
| E_GAP_ | 5,5203 | 5,5367 | 5,5203 | 5,5383 | 5,5386 | 5,5394 |
| *ƞ* | 2,7602 | 2,7683 | 2,7602 | 2,7691 | 2,7693 | 2,7697 |
| *μ* | -3,6160 | -3,6441 | -3,6160 | -3,6536 | -3,6562 | -3,6604 |
| *χ* | 3,6160 | 3,6441 | 3,6160 | 3,6536 | 3,6562 | 3,6604 |
| ORCon |  |  |  |  |  |  |
| E_HOMO_ | -6,3729 | -6,4097 | -6,4668 | -6,4674 | -6,4709 | -6,4761 |
| E_LUMO_ | -0,8425 | -0,8719 | -0,8978 | -0,8983 | -0,9008 | -0,9046 |
| E_GAP_ | 5,5304 | 5,5377 | 5,5690 | 5,5690 | 5,5701 | 5,5715 |
| *ƞ* | 2,7652 | 2,7689 | 2,7845 | 2,7845 | 2,7851 | 2,7857 |
| *μ* | -3,6077 | -3,6408 | -3,6823 | -3,6828 | -3,6858 | -3,6903 |
| *χ* | 3,6077 | 3,6408 | 3,6823 | 3,6828 | 3,6858 | 3,6903 |

Table S3. CT parameters for the bright low-lying excited state for benzofuran derivatives

|  | NH_2_ | | NHR | | NO_2_ | | OH | | OR | |
| --- | --- | --- | --- | --- | --- | --- | --- | --- | --- | --- |
|  | *q*_CT_ | *D*_CT_ | *q*_CT_ | *D*_CT_ | *q*_CT_ | *D*_CT_ | *q*_CT_ | *D*_CT_ | *q*_CT_ | *D*_CT_ |
| THF | 0.744 | 1.118 | 0.743 | 1.111 | 1.136 | 3.341 | 0.743 | 1.148 | 0.744 | 1.096 |
| MeAc | 0.744 | 1.144 | 0.744 | 1.142 | 1.135 | 3.414 | 0.743 | 1.156 | 0.743 | 1.144 |
| MeCN | 0.744 | 1.152 | 0.744 | 1.152 | 1.136 | 3.422 | 0.743 | 1.166 | 0.743 | 1.155 |
| DMF | 0.744 | 1.152 | 0.743 | 1.153 | 1.136 | 3.423 | 0.743 | 1.166 | 0.743 | 1.155 |
| DMSO | 0.744 | 1.155 | 0.743 | 1.155 | 1.136 | 3.428 | 0.743 | 1.168 | 0.743 | 1.158 |
| Water | 0.744 | 1.158 | 0.743 | 1.159 | 1.136 | 3.438 | 0.743 | 1.171 | 0.743 | 1.163 |

Table S4. CT parameters for the bright low-lying excited state for conjugates

|  | NH_2_Con | | NHRCon | | NO_2_Con | | OHCon | | ORCon | |
| --- | --- | --- | --- | --- | --- | --- | --- | --- | --- | --- |
|  | q_CT_ | D_CT_ | q_CT_ | D_CT_ | q_CT_ | D_CT_ | q_CT_ | D_CT_ | q_CT_ | D_CT_ |
| THF | 0.451 | 1.108 | 0.451 | 1.216 | 1.167 | 3.358 | 0.510 | 2.072 | 0.498 | 1.910 |
| MeAc | 0.464 | 1.301 | 0.465 | 1.358 | 1.167 | 3.412 | 0.517 | 2.171 | 0.509 | 2.062 |
| MeCN | 0.469 | 1.322 | 0.390 | 1.404 | 1.167 | 3.430 | 0.519 | 2.189 | 0.461 | 1.968 |
| DMF | 0.469 | 1.264 | 0.469 | 1.346 | 1.167 | 3.431 | 0.518 | 2.136 | 0.456 | 1.832 |
| DMSO | 0.470 | 1.275 | 0.470 | 1.364 | 1.167 | 3.412 | 0.518 | 2.150 | 0.470 | 1.935 |
| Water | 0.471 | 1.333 | 0.471 | 1.438 | 1.165 | 3.449 | 0.521 | 2.207 | 0.471 | 2.068 |

Table S5. Free energies (Δ*G*_solv_, kcal/mol) of solvation for benzofuran derivatives

|  | THF | MeAc | MeCN | DMF | DMSO | Water |
| --- | --- | --- | --- | --- | --- | --- |
| NH_2_ | -19.18 | -21.49 | -21.35 | -21.29 | -20.14 | -18.63 |
| NHR | -19.09 | -21.36 | -21.27 | -20.77 | -19.45 | -17.84 |
| NO_2_ | -17.09 | -19.49 | -20.98 | -18.69 | -17.40 | -14.29 |
| OH | -18.41 | -21.41 | -20.87 | -20.54 | -19.36 | -19.30 |
| OR | -17.91 | -20.03 | -19.78 | -19.50 | -18.16 | -16.40 |

Table S6. Free energies (Δ*G*_solv_, kcal/mol) of solvation for conjugates

|  | THF | MeAc | MeCN | DMF | DMSO | Water |
| --- | --- | --- | --- | --- | --- | --- |
| NH_2_Con | -19.25 | -21.96 | -21.52 | -20.70 | -19.26 | -19.10 |
| NHRCon | -18.57 | -21.62 | -21.19 | -20.64 | -19.02 | -18.10 |
| NO_2_Con | -16.62 | -17.18 | -18.30 | -17.90 | -16.29 | -13.55 |
| OHCon | -18.28 | -20.65 | -20.65 | -19.91 | -18.48 | -19.83 |
| ORCon | -17.95 | -20.40 | -20.40 | -19.81 | -18.14 | -16.96 |

Table S7. The vertical excitation energies (in nm) for benzofuran derivatives

|  | NH_2_ | | NHR | | NO_2_ | | OH | | OR | |
| --- | --- | --- | --- | --- | --- | --- | --- | --- | --- | --- |
|  | λ_ABS_ | *f* | λ_ABS_ | *f* | λ_ABS_ | *f* | λ_ABS_ | *f* | λ_ABS_ | *f* |
| THF | 315.46 | 1.1337 | 315.49 | 0.9649 | 345.05 | 1.0458 | 315.62 | 0.9274 | 315.58 | 1.0216 |
| MeAc | 314.41 | 1.1228 | 314.45 | 0.7383 | 344.73 | 1.0460 | 314.59 | 1.0074 | 314.55 | 1.0565 |
| MeCN | 314.14 | 1.1174 | 314.33 | 0.7589 | 344.65 | 1.0452 | 314.47 | 1.0722 | 314.29 | 1.0621 |
| DMF | 314.18 | 1.1642 | 314.21 | 0.8030 | 344.62 | 1.0476 | 314.36 | 1.0685 | 314.33 | 1.1120 |
| DMSO | 314.10 | 1.1582 | 314.14 | 0.7520 | 344.59 | 1.0474 | 314.29 | 1.0649 | 314.25 | 1.1090 |
| Water | 313.94 | 1.1132 | 313.98 | 0.7062 | 344.42 | 1.0461 | 314.12 | 1.0190 | 314.09 | 1.0666 |

Table S8. The cLR corrected excitation energies (in nm) for benzofuran derivatives

|  | NH_2_ | NHR | NO2 | OH | OR |
| --- | --- | --- | --- | --- | --- |
| THF | 318.83 | 318.83 | 342.75 | 318.98 | 318.91 |
| MeAC | 317.67 | 317.68 | 340.09 | 317.84 | 317.78 |
| MeCN | 317.34 | 317.57 | 338.98 | 317.53 | 317.46 |
| DMF | 317.92 | 317.93 | 337.29 | 318.11 | 318.04 |
| DMSO | 317.77 | 317.80 | 337.29 | 317.96 | 317.90 |
| Woda | 317.10 | 317.12 | 338.14 | 317.28 | 317.23 |

Table S9. The vertical excitation energies (in nm) for conjugates

|  | NH_2_Con | | NHRCon | | NO_2_Con | | OHCon | | ORCon | |
| --- | --- | --- | --- | --- | --- | --- | --- | --- | --- | --- |
|  | λ_ABS_ | *f* | λ_ABS_ | *f* | λ_ABS_ | *f* | λ_ABS_ | *f* | λ_ABS_ | *f* |
| THF | 259.94 | 1.2846 | 259.83 | 1.2708 | 373.06 | 1.0428 | 261.98 | 1.3046 | 261.47 | 1.3024 |
| MeAc | 259.66 | 1.2753 | 259.67 | 1.2705 | 371.87 | 1.0433 | 260.70 | 1.2825 | 260.77 | 1.2842 |
| MeCN | 259.42 | 1.2823 | 259.60 | 1.0350 | 371.43 | 1.0435 | 260.51 | 1.2780 | 259.97 | 1.1729 |
| DMF | 259.72 | 1.3008 | 259.79 | 1.2853 | 371.55 | 1.0447 | 260.81 | 1.2957 | 260.18 | 1.1936 |
| DMSO | 259.66 | 1.2991 | 259.74 | 1.2827 | 371.41 | 1.0446 | 260.73 | 1.2930 | 260.13 | 1.1873 |
| Water | 259.34 | 1.2824 | 259.44 | 1.2661 | 371.08 | 1.0435 | 260.39 | 1.2749 | 259.90 | 1.1615 |

Table S10. The cLR corrected excitation energies (in nm) for conjugates

|  | NH_2_Con | NHRCon | NO_2_Con | OHCon | ORCon |
| --- | --- | --- | --- | --- | --- |
| THF | 259.11 | 259.15 | 373.03 | 261.52 | 260.93 |
| MeAC | 259.14 | 259.15 | 368.60 | 260.77 | 260.74 |
| MeCN | 258.93 | 274.35 | 366.80 | 260.69 | 260.18 |
| DMF | 259.02 | 259.26 | 367.38 | 260.84 | 260.17 |
| DMSO | 258.97 | 259.25 | 365.85 | 260.81 | 260.23 |
| Woda | 258.88 | 259.14 | 365.39 | 260.64 | 260.37 |

Table S11. Calculated values of dipole moments (in D) for the ground and CT excited state fo benzofuran derivatives

|  | NH_2_ | | NHR | | NO_2_ | | OH | | OR | |
| --- | --- | --- | --- | --- | --- | --- | --- | --- | --- | --- |
|  | μ_GS_ | μ_CT_ | μ_GS_ | μ_CT_ | μ_GS_ | μ_CT_ | μ_GS_ | μ_CT_ | μ_GS_ | μ_CT_ |
| THF | 8.87 | 16.85 | 8.80 | 16.59 | 4.87 | 15.08 | 8.74 | 16.83 | 9.18 | 17.32 |
| MeAc | 9.29 | 16.69 | 9.22 | 17.20 | 5.13 | 15.33 | 9.08 | 17.36 | 9.54 | 17.83 |
| MeCN | 9.41 | 16.47 | 9.30 | 17.08 | 5.18 | 15.38 | 9.14 | 17.42 | 9.64 | 18.00 |
| DMF | 9.41 | 16.39 | 9.35 | 16.97 | 5.21 | 15.40 | 9.20 | 17.56 | 9.65 | 18.01 |
| DMSO | 9.44 | 16.34 | 9.38 | 16.92 | 5.23 | 15.41 | 9.23 | 17.62 | 9.68 | 18.05 |
| Water | 9.50 | 16.31 | 9.43 | 16.89 | 5.26 | 15.44 | 9.28 | 17.69 | 9.72 | 18.12 |

Table S12. Calculated values of dipole moments (in D) for the ground and CT excited state for conjugates

|  | NH_2_Con | | NHRCon | | NO_2_Con | | OHCon | | ORCon | |
| --- | --- | --- | --- | --- | --- | --- | --- | --- | --- | --- |
|  | μ_GS_ | μ_CT_ | μ_GS_ | μ_CT_ | μ_GS_ | μ_CT_ | μ_GS_ | μ_CT_ | μ_GS_ | μ_CT_ |
| THF | 7.41 | 13.16 | 7.47 | 13.19 | 2.44 | 21.56 | 6.31 | 11.36 | 6.76 | 13.32 |
| MeAc | 7.79 | 13.34 | 7.89 | 13.89 | 2.45 | 21.53 | 6.68 | 12.67 | 7.14 | 12.44 |
| MeCN | 7.92 | 13.86 | 7.97 | 13.96 | 2.44 | 21.62 | 6.78 | 12.69 | 5.54 | 11.18 |
| DMF | 7.93 | 14.13 | 8.00 | 14.16 | 2.44 | 21.63 | 6.78 | 12.78 | 5.54 | 11.25 |
| DMSO | 7.96 | 14.14 | 8.04 | 14.17 | 2.44 | 21.65 | 6.81 | 12.79 | 5.56 | 11.19 |
| Water | 8.01 | 13.91 | 8.08 | 13.91 | 2.43 | 21.68 | 6.86 | 12.70 | 5.58 | 11.02 |

Table S13. The vertical de-excitation energies (in nm) for benzofuran derivatives

|  | NH_2_ | NHR | NO_2_ | OH | OR |
| --- | --- | --- | --- | --- | --- |
| THF | 401.62 | 401.64 | 446.69 | 401.9 | 401.95 |
| MeAc | 397.95 | 397.99 | 443.65 | 398.29 | 398.28 |
| MeCN | 397.01 | 397.04 | 436.15 | 397.37 | 397.34 |
| DMF | 397.02 | 397.05 | 436.05 | 397.38 | 397.36 |
| DMSO | 396.77 | 396.78 | 435.47 | 397.12 | 397.09 |
| Water | 396.32 | 396.33 | 434.56 | 396.67 | 396.64 |

Table S14. The vertical de-excitation energies (in nm) for conjugates

|  | NH_2_Con | NHRCon | NO_2_Con | OHCon | ORCon |
| --- | --- | --- | --- | --- | --- |
| THF | 362.47 | 362.49 | 429.69 | 362.75 | 362.80 |
| MeAc | 358.51 | 358.55 | 427.09 | 358.85 | 358.84 |
| MeCN | 357.90 | 357.93 | 415.82 | 358.26 | 358.23 |
| DMF | 357.65 | 357.68 | 414.85 | 358.01 | 357.99 |
| DMSO | 357.50 | 357.51 | 414.24 | 357.85 | 357.82 |
| Water | 356.88 | 356.89 | 412.67 | 357.23 | 357.20 |

Table S15. Nonlinear optical properties for benzofuran derivatives. Values are given in a.u.

|  | NH_2_ | | NHR | | NO_2_ | | OH | | OR | |
| --- | --- | --- | --- | --- | --- | --- | --- | --- | --- | --- |
|  | $\left\langle\alpha\right\rangle$ | $\beta_{vec}$ | $\left\langle\alpha\right\rangle$ | $\beta_{vec}$ | $\left\langle\alpha\right\rangle$ | $\beta_{vec}$ | $\left\langle\alpha\right\rangle$ | $\beta_{vec}$ | $\left\langle\alpha\right\rangle$ | $\beta_{vec}$ |
| THF | 200.58 | 546.28 | 215.60 | 499.40 | 210.43 | 404.04 | 195.34 | 575.99 | 210.92 | 514.119 |
| MeAc | 208.91 | 629.04 | 224.64 | 574.35 | 219.34 | 486.56 | 203.25 | 667.76 | 219.44 | 598.19 |
| MeCN | 211.18 | 652.10 | 225.39 | 581.29 | 221.77 | 509.27 | 205.44 | 693.50 | 221.77 | 621.29 |
| DMF | 211.30 | 653.42 | 227.25 | 596.18 | 221.91 | 510.59 | 205.56 | 695.00 | 221.90 | 622.60 |
| DMSO | 211.93 | 659.79 | 227.95 | 601.66 | 222.58 | 517.19 | 206.16 | 702.26 | 222.54 | 628.95 |
| Water | 212.91 | 669.93 | 228.99 | 610.60 | 223.63 | 526.91 | 207.11 | 713.63 | 223.56 | 639.03 |

Table S16 Nonlinear optical properties for conjugates. Values are given in a.u.

|  | NH_2_Con | | NHRCon | | NO_2_Con | | OHCon | | ORCon | |
| --- | --- | --- | --- | --- | --- | --- | --- | --- | --- | --- |
|  | $\left\langle\alpha\right\rangle$ | $\beta_{vec}$ | $\left\langle\alpha\right\rangle$ | $\beta_{vec}$ | $\left\langle\alpha\right\rangle$ | $\beta_{vec}$ | $\left\langle\alpha\right\rangle$ | $\beta_{vec}$ | $\left\langle\alpha\right\rangle$ | $\beta_{vec}$ |
| THF | 219.61 | 1.08 | 234.68 | 1.28 | 229.65 | 3.32 | 214.47 | 0.47 | 229.96 | 0.79 |
| MeAc | 228.67 | 0.86 | 244.17 | 1.12 | 239.30 | 3.30 | 223.21 | 0.23 | 239.25 | 0.56 |
| MeCN | 231.18 | 0.80 | 251.63 | 2.92 | 241.96 | 3.32 | 225.62 | 0.17 | 241.40 | 1.82 |
| DMF | 231.33 | 0.80 | 247.32 | 1.06 | 242.11 | 3.31 | 225.76 | 0.17 | 241.55 | 1.82 |
| DMSO | 232.02 | 0.78 | 248.06 | 1.05 | 242.85 | 3.32 | 226.43 | 0.15 | 242.25 | 1.85 |
| Water | 233.11 | 0.75 | 249.23 | 1.02 | 244.02 | 3.31 | 227.49 | 0.12 | 243.36 | 1.88 |

Table S17. Biological activities for benzofuran derivatives

|  | NH_2_ | NHR | NO_2_ | OH | OR |
| --- | --- | --- | --- | --- | --- |
| Alpha-Radioprotector activity | 0.7378 | 0.0425 | 0.9733 | 0.6335 | 0.8906 |
| Acyl-CoA-holesterol transferase inhibitory activity | 0.4980 | 0.9789 | 0.5628 | 0.2818 | 0.7802 |
| Adrenoreceptor inhibitory activity Anti-Hypertensive activity | 0.0601 | 0.2316 | 0.0000 | 0.0010 | 0.0012 |
| Alpha-R-receptor inhibitory activity_ | 0.0000 | 0.0000 | 0.0000 | 0.0000 | 0.0000 |
| Gamma-radioprotector activity mechanism I | 0.8616 | 0.6167 | 0.8834 | 0.6149 | 0.0003 |
| Analgetic activity | 0.0000 | 0.0000 | 0.0518 | 0.0000 | 0.0000 |
| Anti Awesky Desease activity | 0.7333 | 0.1639 | 0.8004 | 0.1103 | 0.1094 |
| Anti Crymean Haemorrhagic Fever activity | 0.0000 | 0.0000 | 0.0000 | 0.0000 | 0.0000 |
| Anti Herpes Simplex virus activity | 0.8732 | 0.9068 | 0.4033 | 0.7515 | 0.8807 |
| Anti infectious laryngotracheitis activity | 0.5517 | 0.0000 | 0.0238 | 0.6499 | 0.6495 |
| Anti Issyk-Kul Haemorrhagic Fever activity | 0.8708 | 0.9544 | 0.4496 | 0.6817 | 0.1824 |
| Anti Karelian Fever activity | 0.8012 | 0.9680 | 0.0000 | 0.7197 | 0.876 |
| Anti Rift valley Fever activity | 0.9830 | 0.6202 | 0.8766 | 0.9974 | 0.8604 |
| Anti-Adenovirus activity | 0.0285 | 0.7762 | 0.0635 | 0.0770 | 0.1861 |
| Anti-Arrhytmic activity | 0.4319 | 0.6589 | 0.0044 | 0.0008 | 0.3061 |
| Anti-Bacterial activity | 0.9298 | 0.9192 | 0.0000 | 0.7261 | 0.7261 |
| Anti-Oxidant activity | 0.0000 | 0.0619 | 0.5719 | 0.0036 | 0.0000 |
| Anti-Psychotic activity diazepine site | 0.4156 | 0.8440 | 0.5548 | 0.5715 | 0.6539 |
| Anti-Tumor Alkylic activity | 0.2539 | 0.5468 | 0.0259 | 0.0395 | 0.0342 |
| Anti-Tumor Antimitotic activity | 0.2429 | 0.3133 | 0.0087 | 0.2134 | 0.4151 |
| Anti-Tumor Cycline-dependent kinase 4 inhibitory activity | 0.0479 | 0.2155 | 0.7166 | 0.3375 | 0.6578 |
| Anti-Tumor Dihydrofolate reductase inhibitory activity | 0.0887 | 0.1222 | 0.0071 | 0.3960 | 0.4273 |
| Anti-Tumor DNA anti-metabolitic activity | 0.7355 | 0.2715 | 0.8990 | 0.9516 | 0.982 |
| Anti-Tumor Topoisomerase I inhibitory activity | 0.9877 | 0.9780 | 1.0000 | 0.9914 | 0.9997 |
| Anti-Tumor Topoisomerase II inhibitory activity | 0.4168 | 0.1222 | 0.0000 | 0.1579 | 0.9552 |
| HIV1-proteaze inhibitory activity | 0.0616 | 0.5500 | 0.6747 | 0.6155 | 0.7422 |
| HT51 A inhibitory activity | 0.0149 | 0.2598 | 0.9844 | 0.1328 | 0.4295 |
| Tuberculostatic Dihydrofolate reductase inhibitory activity | 0.1108 | 0.9185 | 0.0000 | 0.0308 | 0.8960 |
| Human factor XA Inhibitory activity | 0.0000 | 0.0000 | 0.0000 | 0.3726 | 0.0000 |
| Metabolism at CYP450 2D6 | 0.1747 | 0.0000 | 0.0304 | 0.3237 | 0.0614 |
| Metabolism at CYP450 3A4 | 0.0912 | 0.6137 | 0.0543 | 0.0094 | 0.1679 |
| Progestagenic activity | 0.0000 | 0.2168 | 0.0118 | 0.0000 | 0.0000 |
| Vasorelaxant_activity | 0.2991 | 0.6275 | 0.0000 | 0.3203 | 0.0373 |
| COX1 inhibitory activity | 0.8821 | 0.8003 | 0.5746 | 0.3839 | 0.8434 |
| COX2 inhibitory activity | 0.0000 | 0.0000 | 0.0000 | 0.0040 | 0.0000 |

Table S18. Biological activities for conjugates

|  | NH_2_Con | NHRCon | NO_2_Con | OHCon | ORCon |
| --- | --- | --- | --- | --- | --- |
| Alpha-Radioprotector activity | 0.9276 | 0.0425 | 0.9826 | 0.8578 | 0.7875 |
| Acyl-CoA-holesterol transferase inhibitory activity | 0.3085 | 0.5214 | 0.6093 | 0.8524 | 0.0003 |
| Adrenoreceptor inhibitory activity Anti-Hypertensive activity | 0.7451 | 0.0000 | 0.0000 | 0.7571 | 0.9914 |
| Alpha-R-receptor inhibitory activity_ | 0.0981 | 0.2407 | 0.0000 | 0.1114 | 0.0991 |
| Gamma-radioprotector activity mechanism I | 0.0358 | 0.2627 | 0.0169 | 0.6564 | 0.4386 |
| Analgetic activity | 0.9522 | 0.9999 | 0.0000 | 1.0000 | 0.0000 |
| Anti Awesky Desease activity | 0.0000 | 0.0511 | 0.7466 | 0.1542 | 0.0000 |
| Anti Crymean Haemorrhagic Fever activity | 0.0000 | 0.0000 | 0.0000 | 0.0170 | 0.2602 |
| Anti Herpes Simplex virus activity | 0.9440 | 0.0073 | 0.1935 | 0.6910 | 0.8671 |
| Anti infectious laryngotracheitis activity | 0.0000 | 0.0000 | 0.7448 | 0.0000 | 0.0000 |
| Anti Issyk-Kul Haemorrhagic Fever activity | 0.0095 | 0.1890 | 0.0024 | 0.0000 | 0.1824 |
| Anti Karelian Fever activity | 0.0710 | 0.0000 | 0.8229 | 0.0000 | 0.0298 |
| Anti Rift valley Fever activity | 0.9719 | 0.9938 | 0.9087 | 0.9847 | 0.1825 |
| Anti-Adenovirus activity | 0.5228 | 0.0002 | 0.0000 | 0.0000 | 0.1861 |
| Anti-Arrhytmic activity | 0.4748 | 0.9481 | 0.0000 | 0.9974 | 0.7449 |
| Anti-Bacterial activity | 0.2696 | 0.7485 | 0.0000 | 0.4100 | 0.0000 |
| Anti-Oxidant activity | 0.8371 | 0.6972 | 0.9903 | 0.7110 | 0.5720 |
| Anti-Psychotic activity diazepine site | 0.0283 | 0.9704 | 0.6259 | 0.8559 | 0.0000 |
| Anti-Tumor Alkylic activity | 0.1052 | 0.4847 | 0.0853 | 0.3110 | 0.1675 |
| Anti-Tumor Antimitotic activity | 0.8673 | 0.6895 | 0.3113 | 0.4242 | 0.4151 |
| Anti-Tumor Cycline-dependent kinase 4 inhibitory activity | 0.8475 | 0.8580 | 0.8233 | 0.0274 | 0.4862 |
| Anti-Tumor Dihydrofolate reductase inhibitory activity | 0.0413 | 0.1254 | 0.0375 | 0.1869 | 0.0331 |
| Anti-Tumor DNA anti-metabolitic activity | 0.8389 | 0.0000 | 0.9975 | 0.0000 | 0.6808 |
| Anti-Tumor Topoisomerase I inhibitory activity | 0.4755 | 0.9992 | 0.9991 | 0.9339 | 0.7747 |
| Anti-Tumor Topoisomerase II inhibitory activity | | 0.0000 | 0.3635 | 0.0000 | 0.0000 |
| HIV1-proteaze inhibitory activity | 0.0000 | 0.7163 | 0.6159 | 0.7947 | 0.7904 |
| HT51 A inhibitory activity | 0.9126 | 0.8606 | 0.9868 | 0.6777 | 0.9634 |
| Tuberculostatic Dihydrofolate reductase inhibitory activity | 0.7526 | 0.9890 | 0.0144 | 0.7441 | 0.6512 |
| Human factor XA Inhibitory activity | 0.0000 | 0.0000 | 0.0000 | 0.2165 | 0.4970 |
| Metabolism at CYP450 2D6 | 0.0000 | 0.0000 | 0.8374 | 0.0000 | 0.0154 |
| Metabolism at CYP450 3A4 | 0.5730 | 0.7195 | 0.0000 | 0.8013 | 0.2008 |
| Progestagenic activity | 0.9672 | 0.0740 | 0.1967 | 0.0701 | 0.6931 |
| Vasorelaxant_activity | 0.0000 | 0.0000 | 0.0005 | 0.0000 | 0.0086 |
| COX1 inhibitory activity | 0.6167 | 0.0677 | 0.0705 | 0.0000 | 0.0000 |
| COX2 inhibitory activity | 0.0000 | 0.0000 | 0.0046 | 0.0000 | 0.1902 |

Table S19. Binding free energies (Δ*G_b_*, kcal/mol) obtained during AutoDock simulations

| Lysine | NH_2_ | NHR | NO_2_ | OH | OR |
| --- | --- | --- | --- | --- | --- |
| LYS30 | -3.1 | -3.1 | -3.0 | -3.0 | -3.1 |
| LYS35 | -4.1 | -4.1 | -3.7 | -3.8 | -4.0 |
| LYS36 | -3.7 | -3.8 | -3.6 | -3.7 | -3.6 |
| LYS39 | -3.4 | -3.1 | -3.4 | -3.5 | -3.3 |
| LYS46 | -3.8 | -3.9 | -3.9 | -3.8 | -3.9 |
| LYS59 | -3.7 | -3.6 | -3.5 | -3.7 | -3.4 |
| LYS101 | -3.9 | -4.2 | -4.0 | -3.9 | -3.9 |
| LYS114 | -5.0 | -4.8 | -5.4 | -5.0 | -4.5 |
| LYS116 | -4.4 | -4.3 | -4.3 | -4.4 | -4.2 |
| LYS135 | -3.7 | -3.3 | -3.5 | -3.6 | -3.3 |
| LYS138 | -4.2 | -4.0 | -3.8 | -4.2 | -3.8 |
| LYS200 | -3.3 | -3.5 | -3.7 | -3.2 | -3.6 |
| LYSTer | -3.9 | -3.8 | -4.0 | -4.0 | -3.7 |
